# Ethical considerations in prehospital ambulance based research: qualitative interview study of expert informants

**DOI:** 10.1186/s12910-019-0425-3

**Published:** 2019-11-27

**Authors:** Stephanie Armstrong, Adele Langlois, Niroshan Siriwardena, Tom Quinn

**Affiliations:** 1 School of Health and Social Care, University of Lincoln, Brayford Pool, Lincoln, LN6 7TS UK; 2 School of Social and Political Science, University of Lincoln, Brayford Pool, Lincoln, LN6 7TS UK; 3 Professor of Primary and Pre-hospital Healthcare, Community and Health Research Unit School of Health and Social Care, University of Lincoln, Brayford Pool, Lincoln, LN6 7TS UK; 4 Emergency, Cardiovascular and Critical Care Research Group, Kingston University and St George’s, University of London, 6th Floor, Hunter Wing Cranmer Terrace, London, SW17 0RE UK

**Keywords:** United Kingdom, Ambulance, Clinical trials, Ethics, Consent, Expert interviews, Paramedics

## Abstract

**Background:**

Prehospital ambulance based research has unique ethical considerations due to urgency, time limitations and the locations involved. We sought to explore these issues through interviews with experts in this research field.

**Methods:**

We undertook semi-structured interviews with expert informants, primarily based in the UK, seeking their views and experiences of ethics in ambulance based clinical research. Participants were questioned regarding their experiences of ambulance based research, their opinions on current regulations and guidelines, and views about their general ethical considerations. Participants were chosen because they were actively involved in, or in their expert capacity (e.g. law) expressed an interest in, ambulance based research.

**Results:**

Fourteen participants were interviewed including principal investigators, researchers, ethicists and medical lawyers. Five major themes were identified: Capacity, Consent, Clinical Considerations, Consultation and Regulation. Questions regarding consent and capacity were foremost in the discussions as all participants highlighted these as areas for concern. The challenges and use of multiple consent models reflected the complexity of research in this environment. The clinical theme referred to the role of paramedics in research and how research involving ambulance services is increasingly informing improvements to patient care and outcomes and reducing the burden on hospital services. Most felt that, although current regulations were fit for purpose, more specific guidance on implementing these in the ambulance setting would be beneficial. This related closely to the theme of consultation, which examined the key role of ethics committees and other regulatory bodies, as well as public engagement.

**Conclusions:**

By interviewing experts in research or ethics in this setting we were able to identify key concerns and highlight areas for future development such as improved guidance.

## Background

Trials involving ambulance services are a rapidly developing field, with increasing numbers and scale of studies globally. Early intervention, for example in myocardial infarction, stroke or cardiac arrest, could significantly improve patient outcomes, but needs to be tested in situ, as existing treatments administered in this context are often either unsatisfactory [1–3] or lack an evidence base [4, 5]. In the UK the types of research undertaken within the ambulance service include drugs trials, device trials and investigations into alternative care pathways. These trials can be complex and several papers identify the unique challenges of prehospital research, both ethical and practical [6–8]. This study sought to identify these challenges in depth with a range of expert stakeholders.

A systematic review of prehospital trials highlighted capacity and consent as being particularly challenging [9]. Patients in the prehospital setting are often incapable of giving consent and are therefore a vulnerable population [3, 10–12]. Even if not unconscious, patients may be in too much pain or be too distressed and overwhelmed by the situation to give the question of consent due consideration [8, 11, 13]. Ambulance-based research also differs from other emergency research settings due to the unpredictability of the location. Incidents could occur in public spaces, patients’ homes or the ambulance itself, for example, all of which might inhibit a patient’s ability to engage with a consent process. Furthermore, time pressures mean that lengthy processes for assessing capacity could be impracticable [8]. Increasingly studies seek to proceed without consent when consent processes would delay an intervention to the extent that it would no longer replicate practice, or when delayed treatment might cause additional stress or harm [1, 11, 14–17].

Due to the complexity of the setting, alternative consent models have been proposed [9]. Surrogate or proxy consent is a common approach whereby the surrogate is asked to act in the ‘best interests’ of the patient [8, 11, 18]. Most often the surrogate will be a relative or close personal friend, but there are provisions that allow a medical professional to act as a proxy. Previously, it has been questioned whether a paramedic or emergency medical technician (EMT) can fulfil this role [17]. Another common option is emergency waiver of consent (sometimes termed ‘delayed consent’ or ‘deferred consent’) [9]. In this model patients are given the intervention without prior consent, and later approached to give consent for ongoing participation and use of data [14]. This is particularly relevant where timeliness of treatment may render it impossible to seek surrogate consent [4, 12, 16, 19]. Studies in both the US and UK found that, whilst most patients felt this form of enrolment was acceptable, some expressed concern over the loss of autonomy that deferred consent entails [15, 20].

In England and Wales clinical research is governed by two pieces of legislation. The Medicine for Human Use (Clinical Trials) Regulation (2004) (MHU-CT) must be adhered to in clinical trials of investigational medicinal products (CTIMP) [21]. The Mental Capacity Act (2005) (MCA) applies to all other research, including trials using non-medicinal interventions such as devices or non-interventional studies [22]. Both pieces of legislation give guidance regarding the enrolment of participants on to trials and in particular those who lack capacity to consent [23]. Ambiguity regarding the correct application of the legislation, especially with regards to participants who have reduced capacity, can be challenging and has been highlighted as an aspect in need of clarification in prehospital research [16].

The complexity of enrolling patients into research in the prehospital setting notwithstanding, it should be remembered that consent is not the only ethical consideration. Paramedics’ traditional priorities of getting patients to hospital as soon as possible whilst providing optimal care may not easily align with research [6, 24–26]. A questionnaire study with UK paramedics found that lack of time, support and opportunities for research also prevented participation [24]. Stronger engagement by prehospital researchers with not only paramedics [6], but also research ethics committees [10, 16] and affected communities [27], can help to foster acceptance of emergency research.

This project sought the views of experts in the field of prehospital ambulance based research, in order to understand the ethical considerations and pressures faced by researches currently working in this setting. Throughout this paper we use the generic term trial(s), except where it is appropriate to specify the type of research undertaken.

## Method

### Participant recruitment

Participants were recruited using a purposeful snowball sampling technique, which allowed us to reach a range of participants through existing contacts. Since we wanted to gain the views of people who had experience in undertaking ambulance based research, we specifically targeted participants who were leading or actively involved in ambulance trials or medical ethics research on emergency or ambulance studies. The initial participants were those who had previously expressed an interest in the ethics of ambulance based trials at a networking event on 1st March, 2016 (Newcastle, UK). Additional participants were identified through recommendations made during the interviews or through contacts at subsequent conferences and meetings. Participants were provided with a participant information sheet and consent form and written consent was gained prior to participation.

### Data collection and analysis

The interviews continued until data saturation was attained [28]. Data were collected through semi-structure interviews either via the telephone or Skype and were digitally recorded. Skype interviews were offered in the first instance, as these have the advantage of both face-to-face interviews, in terms of interaction with the participant, and telephone interviews in terms of convenience and cost savings. The interviews lasted between 25 and 50 min. Approximately half of the interviews were carried out using Skype. Telephone interviews were recorded using a digital Dictaphone whilst Skype interviews were recorded using Audacity software (Free Software Foundation, Boston, USA). Audacity only records audio from the interview, and files are saved in the same digital format as the Dictaphone files. All recordings were transcribed verbatim as soon as possible after the interview and the digital recordings immediately deleted. Participants were asked about their experiences of prehospital trials, the ethical considerations, barriers and facilitators in such trials and consent processes.

The transcriptions were analysed thematically using NVivo 10 and coded independently by two authors (SA and AL). The initial list of codes were agreed through discussions between the coders. These codes were then compared and grouped into higher order themes through discussions within the team (SA, AL and ANS).

### Findings

Fourteen participants were interviewed, 13 based in the UK and one in Sweden. Table 1 lists the participants identifying number alongside their role/research experience. Most of the participants had academic roles, principal researchers generally holding professorial titles, whilst the ethicists and the medical lawyer were also in academic research positions. The research paramedics and research nurse were all in clinical positions with additional research responsibilities.

**Table 1:** List of participants job roles

Code	Experience
EX01	Prehospital principal researcher
EX02	Prehospital principal researcher
EX03	Prehospital research fellow
EX04	Prehospital principal researcher
EX05	Ethicist
EX06	Research paramedic
EX07	Emergency Department researcher
EX08	Prehospital principal researcher
EX09	Prehospital principal researcher
EX10	Research paramedic
EX11	Ethicist
EX12	Emergency Department research nurse
EX13	Prehospital principal researcher
EX14	Medical lawyer

The thematic analysis of the transcriptions resulted in five major themes being identified. These were: capacity, consent, clinical considerations, consultation and regulation. A sixth theme of ethical complexity was pervasive across these five themes.

### Understanding capacity to consent to research (capacity)

The first theme addresses physical and mental capacity and the ability of patients in the ambulance setting to make informed choices. Throughout all the interviews it was apparent that mental capacity was an important ethical consideration in both the design and implementation of ambulance based research. Discussion regarding capacity centred on whether someone who calls an ambulance has the mental capacity to make an informed choice, specifically regarding participation in a trial, even if conscious (EX02, EX03, EX04, EX11, EX12). For example, a prehospital research fellow commented:

Fundamentally I just question this whole concept of consent under those situations for research. I just don’t think it really exists and that the nearest you can get to somebody’s individual consent in those particular circumstances is probably a gut feeling from them. Or a gut feeling from their family about whether they would generally speaking want to take part in research or not. **[EX03].**

And speaking particularly about stroke, a research nurse raised the issue of both mental and physical capacity:

I would strongly question whether anybody who is having a stroke or has had a stroke can make any sort of informed consent decision … ..but then I suppose if you are having a stroke as well it’s how you convey that you can understand that information, if your speech is already gone. **[EX12].**

Assessment of capacity was also a factor. Several participants discussed whether it was possible to assess capacity in the ambulance (EX04, EX07, EX08, EX09, EX11, EX12). One principal researcher suggested the following protocol:

To determine capacity in the ambulance, which was basically telling the patient what their condition was, i.e. stroke, a particular issue which was relevant to them, which was their blood pressure was higher than ideal and the intervention which was a skin patch, which might lower blood pressure. And we then asked them, “What do we think’s wrong with you?” And they were obviously meant to say stroke. And “What’s particularly the issue right now?” – “I’ve got high blood pressure”. “And what do we want to do about it?” – “You wanna put a patch on”. And if they could get those 3 ideas, and relay them back to us then we assumed they had capacity. **[EX09].**

But others countered this by saying that in their experience, even if the patient was able to answer questions at the time, thereby giving consent, they often did not retain this information, even a short time later (EX04, EX07).

Finally, related to this theme were questions about whether the emergency setting may mean that people may be too distressed to really take in the information given to them, or may agree to something when they might not have done had they had more time to consider it (EX03, EX06, EX08, EX10, EX11, EX12). A paramedic voiced their doubts as follows:

… their whole world can change within 10 min. So if you’re now trying to reassure them, address their problems and now you are going to approach them about a research project that actually they need to fully understand the information, the risks, the benefits. Are they necessarily in the right frame of mind to do that? **[EX10].**

And an ethicist commented:

And the third criterion is lack of voluntariness or lack of coercion. And you could argue that that criterion can’t be met in this context, because they are having an emergency event, so they are going to do whatever the hell you suggest. **[EX11].**

This theme raised questions regarding what constitutes capacity in the out-of-hospital emergency setting, the different mental and physical barriers to capacity and how to assess these in the necessarily short time period of the event. These are crucial factors on determining appropriate consent models in ambulance based research.

### Models of consent (consent)

The consent theme contained the largest number of individual codes and covers all aspects of consent in the ambulance research setting. This was the area that generated most discussion, with a wide range of views expressed regarding best practice for gaining consent in ambulance trials. All participants agreed that consent in this setting is complex, particularly in light of the previous discussions regarding capacity. Participants from across a range of roles agreed that gaining prior written informed consent in the ambulance could be problematic (EX05, EX07, EX08, EX10, EX12), and therefore discussion tended to centre on consent methods that could be used where there was a lack of capacity.

Within both the MHU-CT and the MCA there are provisions that allow participants who are unable to give consent and have no surrogate present to be entered into an emergency trial. Participants discussing these provisions often used the terms ‘waived’ or ‘deferred consent’ (EX09, EX10, EX12). For example:

I know that a number of trials are using waiver of consent. I think this absolutely has a place, and certainly where those trials are recruiting patients that cannot consent, they are unconscious, they are in cardiac arrest, there is no consent that can be gained [in] the environment in which they are in the clinical care that they need to provide is timely. **[EX10].**

Waived or deferred consent usually involves a process whereby consent is gained after the emergency has passed (EX05, EX08, EX14). This generally gives the participant (or their representative if they are unconscious [EX08]) the opportunity to withdraw from any further participation in the study or to agree to data use and follow up data collection (EX04, EX09, EX12, EX13):

….so for all of the patients that are enrolled in the emergency situation in A&E we then pursue them and their families throughout their hospital stay and the kind of end goal is to get an informed choice decision, whether that’s to stay in the trial or to leave the trial, from the patient themselves. So that can sometimes be 28 days later, or never if unfortunately they die. **[EX12].**

Proxy or surrogate consent was also discussed in detail in all the interviews. The use of relatives as surrogates, where available, was most common and this was felt to be an acceptable form of consent should a patient be incapacitated. Additionally, some participants discussed whether close friends or work colleagues could act as surrogates (EX05, EX06, EX09, EX11), with one questioning how well a work colleague might know a patient and how to assess this (EX12). The rationale for asking a relative to consent on an incapacitated patient’s behalf is that “it’s the closest that we can get to knowing what the patient’s wishes would have been” (EX03). A principal researcher articulated this thus:

I think that approach that we used is to use a family member as somebody to give consent on behalf of the patient if they don’t have capacity at the time, or at least to give short consent. I think that’s the most appropriate approach for the research that I’ve been involved in. **[EX02].**

A number of participants did express some concerns, regarding whether a relative can accurately represent a patient’s wishes. One said, for example:

I’ve had family members who’ve asked for the patients to be withdrawn from the trial and then when I’ve later gone back and spoken to the patient they’ve said, “Ah no I would’ve stayed in, I would have done it.”… And I’ve had relatives trying to persuade patients to take part in research as well, which is the other way round, which is what you don’t want as well. **[EX12].**

Others agreed that, like patients, potential surrogates can find the emergency too stressful an environment in which to make decisions (EX01, EX06, EX10, EX11:

I think having been out in clinical practice resuscitating people in their houses and in the street and things with family members being very distressed around, that’s not the time to try and have that conversation. **[EX01].**

Finally, some questioned whether anyone but the patient can give ‘consent’ (EX03, EX04). At most, they felt a surrogate could give an opinion (on the patient’s philosophy on health research, for example), rather than consent per se. This is where ambiguity over terminology was apparent in some of the discussions, with several participants using a blanket term of ‘consent’. Note that the MCA uses the language of consultation rather than consent, as surrogates are referred to as ‘consultees’, whereas the MHU-CT allows informed consent by a legal representative.

Under the MHU-CT regulations, if a relative is unavailable, the patient’s doctor or a person nominated by the relevant healthcare provider can be a legal representative and thus able to provide consent, provided they are unconnected with the trial. The use of this type of surrogate was discussed intensely, particularly with regards to paramedics taking this role. One principal researcher described the use of this consent model in a recent CTIMP as “ground breaking in several ways” and “well received by the scientific community” (EX09), whilst a paramedic thought it was acceptable, with the caveat that “the patient or their representatives must always be given consideration and the option to withdraw from the study further down the line” (EX06). Other participants had reservations. The arguments against using paramedics as legal representatives fell into two groups. Firstly, some questioned whether a paramedic was independent of the research (EX01, EX05, EX11, EX13 EX14). For example, one ethicist said:

A nominated legal representative must be an independent doctor or I think – if I remember correctly a person appointed by the trust or health authority for that purpose. So I’ve never been quite sure about who that other person might be, but what it is really saying is that it should be an independent doctor. So I would read from that that a paramedic couldn’t take on the role of the nominated legal representative in a clinical trial. **[EX05].**

Secondly, others felt that it placed undue responsibility on the paramedic during the emergency situation (EX01, EX04, EX08, EX12, EX13). For example, one principal researcher expressed their doubts as follows:

I think it’s placing a lot of emphasis and responsibility onto the paramedic in making that decision and in making that decision when they are highly unlikely to have ever met the patient before or have any basis upon which to make, to form that judgement. **[EX13].**

Due to the complexity of obtaining written consent in ambulance based trials, even from surrogates, other models were discussed. The first was to gain affirmation from the patient that they were happy to proceed. This was termed variously ‘assent’ or ‘short consent’. This model suggests that patients may not be able to give written informed consent because of time pressures or distress, but may be able to consent to the intervention or treatment, and thereby assent to being in the trial (EX02, EX06, EX08, EX10, EX11, EX13). The model can also be used for relatives acting as surrogates (EX03, EX12). The assent would later be confirmed by gaining written consent once the emergency had passed:

We do an oral informed consent first, so the patient gets a very short information about the trial, basically asking, “We are looking at this, are you willing to participate?” And then during the first 24 h of the hospital stay they get a more thorough written informed consent form to read and then to sign. **[EX07].**

Across the expert roles (EX03, EX04, EX05,^1^ EX06, EX13), participants believed that the advantage of this model is that it is workable in the emergency context. For example, one principal researcher commented:

So I think initial assent does make a lot of sense. …I think it better reflects the cognitive capacity of the patients at that particular time. And so I think that model is worthy of exploration but it is not one that is widely recognised. **[EX04].**

Whilst, a paramedic said:

So I think that is a good workable model and does give the patient some input – into their treatment and, given the opportunity, whether they would want to be involved. **[EX06].**

The final model, discussed in some detail, was the idea of ‘opt out’, whereby potential participants receive information prior to the study and are able to opt out of participation by wearing a bracelet or other identification that paramedics must check for. On the whole, those who discussed this option felt that the burden on trials were too great, in terms of both costs and time, to ensure that members of the public had adequate opportunity to opt out (EX02, EX11, EX13).

Furthermore, one researcher described how studies that had used an opt-out system had been “slaughtered” when they presented their data, as the principle of explicit patient consent is so ingrained (EX07).

This theme highlighted that the general challenges to giving consent for trials can be compounded by the ambulance setting. Whilst multiple models for gaining consent exist, it is not always clear when each should be applied.

### Clinical staff involvement in ambulance research (clinical)

The clinical theme refers to the use of paramedics in trials, and how involvement in ambulance based research informs paramedics’ professional competencies, as well as expectations around patient care. The theme is concerned with what the practical barriers to implementation of ethical research in this clinical environment might be. Some participants discussed the relative newness of research in this setting, and the need to ensure that paramedics understand the purpose and processes of research and research ethics. Several participants highlighted the need for general research training for all paramedics, including consent procedures (EX01, EX03, EX04, EX09, EX12).

So there are real training issues and that’s a real barrier and of course that’s relevant to your project on, on ethics and, and consent and so on because we must make sure these people understand the ethics of the study, the consent process. So how do we train people is, is a major issue. **[EX09].**

Perceptions of paramedics’ attitudes to both research and training differed. One prehospital researcher had encountered reluctance on the part of paramedics to engage in research:

There are a significant minority of paramedics who simply don’t want to do research. The worst thing in research is to force people ‘cause they simply don’t do it. You can train them all and they won’t do it. They’ll always have a reason why they didn’t do it, so it’s just a waste of time and effort and it stresses them and so on. **[EX09].**

Others had found paramedics to be enthusiastic about research:

The paramedics were happy to be trained, they were happy to take on that extra responsibility… We’ve had very few paramedics refuse to help with the study. **[EX03].**

So I think from paramedics we’ve met a lot of enthusiasm and support for important research questions. **[EX13].**

The two research paramedics noted that paramedics can be unfamiliar with the research context and ethical approval procedures, as well as uncertain about whether research is in patients’ best interests:

Assessing the capacity of that participant or potential participant could be quite daunting the first time you come to do it. And without any sort of research experience that can be quite difficult. **[EX10].**

Is it ethical to involve people in trials if it delays their treatment is one of the big questions that we get asked. **[EX06].**

Yet they and others (EX03, EX04, EX09) recognised that the research culture within ambulance services is already showing signs of change, as more paramedics are now trained at degree level and thus “exposed to research, exposed to evidence based medicine from day one” (EX06). Prehospital research would thus be “missing a trick” if it did not use paramedics (EX10).

Despite these changes, the challenges of developing a research culture in a relatively research-naïve organisation (i.e. the ambulance service), and the difficulties of disseminating information throughout this organisation, were also raised:

I think whilst there has been such a wealth of research that’s been done so far it’s, it, there’s always been difficulty in disseminating that out to staff members. And if you go and talk to a standard road paramedic about what research is going on, if they don’t actively have that interest, I think the communication and the spread of the knowledge hasn’t really reached its full potential. **[EX10].**

In this regard, peer education and engaging paramedics in the development of an intervention have proved invaluable in fostering ownership of research among paramedics (EX01, EX03).

The future of ambulance based trials and the need for ambulance organisations to prioritise research, were also discussed by both principal researchers (EX01, EX04) and the two research paramedics (EX06, EX10). For example:

I think we need to get better at actually integrating research and making [it] an organisational priority. Most ambulance services are really building their research teams and the level of research that’s going on is developing all the time. But I think there’s some work we can do with staff on the ground who are now equipped with the research skills – let’s use them a bit more and involve them. **[EX10].**

In general, it was felt that, whilst barriers to research in ambulance services exist, especially in the form of organisational priorities and continued research naïvety, ambulance research was expanding and would provide some of the next advances in health care, as summarised by one participant:

In the context of stroke the ambulance prehospital environment is the next great clinical laboratory for doing this sort of research. **[EX09].**

### Consultation with ethics committees and public involvement (consultation)

Lack of experience of ambulance based trials was also evident in the consultation theme, which deals mainly with ethical review and the role of ethics committees in research, alongside wider consultation. There was extensive discussion by participants regarding the role of ethics committees. UK-based principal researchers, paramedics, and ethicists alike felt that ethics committees varied widely in their understanding of the ethical issues related specifically to ambulance trials, particularly in terms of the unique consent considerations and patient environment, which led to inconsistent responses to applications for ethical approval (EX01, EX04, EX05, EX06, EX08, EX10). Two principal researchers said:

The ethics committees within the UK are very variable in their response to this kind of problem. Some of them are well informed and helpful and sometimes they are so well informed in fact they are quite positive around research without consent. Sometimes they are extremely unhelpful. **[EX04]**

...and the inconsistency of decisions that you get from them. So both from the ethics committees and from individual participating research sites. They can end up completely tied in knots And it changes from trial to trial, from ethics committee to ethics committee, from site to site. **[EX08].**

There has been similar inconsistency in Sweden, where some ethics committees have approved oral assent followed by later written consent, whilst others have insisted on prior written consent. There is also “a lot of responsibility if something goes wrong” for an ethics committee, which may lead to caution over opt-out trials, for example (EX07). The variability in the UK was put down to the newness of the field by one paramedic:

Prehospital research is very new to ethics committees and most ethics committees may not have people with any experience or knowledge of prehospital studies. **[EX06].**

Thus ways forward may include setting up specialist committees and specific guidance and training for committees (EX02, EX04, EX06, EX08).

Where this has already happened through the National Research Ethics Service (NRES),^2^ this has “made life easier all round and more efficient because you had a body of people round specialist ethics committees who understood the regulations and were used to dealing with the complexity of these very vulnerable patients” (EX01).

According to the two paramedics (EX06, EX10), another option is to invite those with prehospital research experience on to existing ethics committees:

Including somebody on the ethics committee with prehospital background, experience, knowledge, dedicated insight into that realm, really that could actually provide that specialist knowledge for the types of trials that we’re going to be doing. **[EX10].**

In terms of wider consultation, patient and public involvement is increasingly seen as an essential part of the research process, from inception to completion, as one principal researcher attested:

I think public consultation is important. It’s not a requirement of the current legislation that there is public consultation, but I think it’s a marker [of] best practice. And certainly, any NIHR [National Institute for Health Research] funded study would expect, pretty intensive patient involvement. I think it’s right to do so, it’s just I don’t think it’s mandated to do so. **[EX13].**

They described some of their experiences with such consultations. At a meeting with around 300 people in attendance, about 80–90% were in favour of the waived consent model being proposed, but more people supported the research in general terms than said they would be willing to enrol in the trial should they suffer the condition under investigation.^3^ Overall, this researcher saw sharing information with the wider community as a facilitator of research, but barriers included mistrust of the government and the medical profession (EX13).

Other principal researchers also outlined their experiences. One (EX01) stated, “Obviously we have patient and public engagement in the development of these studies and consideration of the ethical aspects”. They described how, on one trial, the ethics committee required the wider public to be informed, so materials were translated into “a whole host of languages” and disseminated via the trial website. It was important that the public had as much information as possible before the point of recruitment, “because at the point of recruitment it doesn’t work because the patient is unconscious and the family are very distressed”. There was also in-depth consultation with a smaller group (5–20 people), comprised of patient representatives (former patients or their partners or children) and “so people who would be even more objective who haven’t actually had exposure to the condition and things and bringing a different view point”. Another (EX02) related how a Patient and Public Involvement (PPI) group on one trial had insisted that the decision they were making was particular to that trial “and that they didn’t want that to be a precedent for all prehospital studies”. They also advocated public consultation beyond PPI.

This theme dealt with the impact of ethics committees and public consultation on prehospital research. Several participants reiterated the importance of guidance, training and engagement with ethics committees and the public more broadly to facilitate understanding of the need for ambulance based research and the ethical considerations involved.

### Regulation within the United Kingdom (regulation)

The regulation theme concerns current legislation governing research in the UK, including the Medicines for Human Use (Clinical Trials) Regulations (2004) and the Mental Capacity Act (2005).^4^ Participants largely agreed that legislation was fit for purpose, although some commented on the challenge of getting to this position (EX04, EX05, EX09):

And so the challenge has been to find mechanisms and to lobby relevant legislative and regulatory bodies to accept the need to try research in incapacitated individuals and to support that research effectively. **[EX04].**

The UK is perhaps more advanced than some other countries in this respect. In Sweden, for example, emergency research is more restricted legally:

The situation in Sweden is like this, that by law you have to ask a patient to participate. If the patient cannot answer, does not understand what you are saying or is too sick to answer, then in principle the patient is not allowed to participate in a trial. That’s obviously a big problem for acute studies. **[EX07].**

Although some Swedish studies have now been allowed to use waived consent, others have not been allowed to enrol unconscious people (EX07).

Whilst the UK legislation was considered adequate, several participants reported that there was often lack of knowledge or confusion over the application of the regulations, even among regulatory agencies (EX01, EX03, EX05, EX08, EX10). Waiver of consent under both the MHU-CT and the MCA must be approved by an NHS (National Health Service) Research Ethics Committee, who have determined that the investigative treatment would be in the patient’s best interest and unlikely to cause harm. Two principal prehospital researchers commented:

I think the amendment in 2006 to the Medicines for Human Use (Clinical Trials) Regulations was very welcome.^5^ … What was unhelpful was the ethics committees didn’t seem to know about those regulations, that statutory instrument. And still a lot of people don’t know about it. I think if you don’t work in this particular world you won’t necessarily know that those regulations exist. **[EX01].**

Our ethics committee had just said it was ethical but we would need information governance approval. And the information governance people said “No this is not ethical”. **[EX08].**

All participants highlighted the need for clear, specific guidance regarding ambulance based research. This would be useful in and of itself, but also to help prehospital researchers in justifying their research plans:

Because it is such a new field, any sort of well-founded guidance would be gratefully received by most people and would give those working in prehospital research something to work to and to support their claims. **[EX06].**

One principal researcher suggested a comprehensive framework that covered a range of scenarios, as current guidance tends to focus on particular trial types (with unconscious patients, for example), which does not work when applied to other trial types:

I think that we have [to] work with the legislation that’s there, but I think there are lots of nuances that … require guidance. … And if we had some comprehensive guidance which covers the various eventualities and the various scenarios that the ethics committees and the information/R&D governance people could refer to we might waste much less time. **[EX08].**

They went on to say that writing useful and accessible guidance is not enough, as it then needs to be implemented (EX08). Adoption might be facilitated by ensuring wide consultation on any guideline revisions, to include ethics committees, researchers and the public, as suggested by one ethicist (EX05).

Any guidance would have to be future-proofed. One principal researcher was concerned that guidelines could become prescriptive in terms of setting out “you can do this and you can’t do this”, rather than providing “guidance for things that need to be considered” (EX02). They went on:

It would be a mistake to set in place things now which would limit the opportunities for innovative research to happen, I guess the experience with research in general makes me slightly cautious about that. **[EX02].**

## Discussion

The findings of this study showed strong support among participants for the principles of biomedical ethics, but also highlighted the ongoing challenges that ambulance based researchers face in operationalising these in the prehospital setting. Research in this relatively new setting is likely to expand, as the need for early and effective healthcare interventions delivered by ambulance clinicians is increasingly prioritised [4]. Both the literature and our findings suggest that, whilst the ambulance setting offers unique opportunities for research, there are specific ethical challenges to be met.

Capacity (both mental and physical) and the ability to gain informed consent were highlighted as problematic in this setting. Participants spoke about patient stress and pain and time limitations in the emergency context, with several stating that consent given in these circumstances may not be meaningful. Whilst these factors may not be unique to the ambulance setting, they are compounded by the nature of a prehospital emergency call, where paramedics often work alone or in pairs and are pressured to complete a call quickly so that they can move on to the next call. This means that using accepted methods of enrolling patients on to trials is difficult. Related to this is the need to communicate complex information regarding a trial in a short space of time, raising concerns regarding the retention of information by distressed patients and relatives. This is reflected in the literature, where similar issues have been discussed [13]. It may be necessary to develop strategies to mitigate these concerns when enrolling patients into trials.

Most interviews included discussions regarding the use of legal representatives and consultees as surrogates for patients who lack capacity, often referred to by the participants as proxy consent. This in turn led to discussions regarding whether a paramedic can act as an independent legal representative for trials of medicinal products under the MHU-CT, as in the ambulance setting all paramedics on scene would be directly involved in patient care, and therefore could not be seen as independent of the trial where part of the care constituted the intervention. The same question does not apply to other types of ambulance based research, as the MCA determines that a consultee who can advise the researcher on the patient’s wishes with regards to participation in research must be sought. As this assumes that the patient will be well known to the consultee, a paramedic could not fulfil the consultee role, as it is unlikely that they would have prior knowledge of the patient.

Alternative methods of gaining consent were also discussed, including waived or deferred consent and verbal assent after a basic explanation of the intervention, followed by later written consent to remain in the study. Emergency waiver of consent is permitted under the UK legislation where the treatment (both medicinal and non-medicinal) is urgent and it is not practicable to seek advice from a consultee (MCA) or a legal representative (MHU-CT). Several of the expert informants considered assent a workable option for ambulance based research. Similarly, in an interview study with UK patients, most participants thought this a viable method (albeit in the form of an abridged information sheet), provided they would receive further information later on [15]. One consent model that garnered particular debate was the use of an advance opt-out system, similar to that required by some institutional review boards (ethics committees) in the US [29]. The reverse – that is prospective opt-in – can be equally problematic. Predicting the need for emergency treatment is difficult and makes it challenging to recruit enough potential participants without incurring huge costs in both time and money. The time-critical nature of such an event also means it may not be possible to verify that a person has prospectively consented without unduly delaying treatment [18].

All participants discussed the need for training and guidance for paramedics and researchers on the ethical challenges of ambulance based trials, in particular where patients are conscious but incapable of giving fully informed consent. With regard to what needs to happen for more paramedics to engage in research, the views and experiences of both principal researchers and paramedics largely corresponded with existing literature. First, paramedics will need assurances that research will not adversely delay treatment or access to hospital for patients [26]. Second, research will need to be seen as a core responsibility of the paramedic role, rather than a voluntary add-on [4, 26]. Third, paramedics will need to be included in the research development process, thus reinforcing their professional identities and acknowledging their expertise [6]. There were similar correlations between the experiences of the UK-based prehospital researchers interviewed and those of their counterparts in the US, in terms of receiving inconsistent or unhelpful decisions from ethics committees [30]. As with paramedics, engaging with ethics committees from the outset, as well as specialised training on prehospital research for committee members, could help to resolve these issues [7, 10, 16]. Prehospital researchers in the UK had, in general, had more positive experiences of public consultation than those in the US, however. Whereas both smaller PPI-type sessions and larger meetings were considered useful by UK-based principal researchers, only the former have proved worthwhile in the US [10, 30]. Finally, whilst current legislation and regulations do allow for the development of models of enrolment and consent appropriate to ambulance based trials, many participants felt these were not well understood. All participants highlighted the need for better guidance on the application of the legislation specific to ambulance based research.

### Strengths and limitations

This interview study offers a unique insight into the ethical challenges of undertaking research in a developing field, the ambulance service. The snowball sampling method was chosen as it was felt this would be the best way to gain buy-in from potential participants in what is a relatively small field. It is important to acknowledge the limitations of this method, which can included possible selection bias [31]. However, it was felt that the advantages of this methods outweighed these potential disadvantages. By interviewing experienced researchers and ethicists we were able to identify areas for concern. Due to the time constraints of the project, this initial study interviewed mainly UK based experts, which limited the analysis to UK regulations and research. We also restricted participation to those who were currently active in, or who had a stated interest in, ambulance based research. It may have been useful to have included paramedics and research ethics committee members not currently engaged in or with prehospital research, as well as patient representatives, to get alternative perspectives, but time constraints did not allow for this.

### Implications for further research

Our research poses a number of questions regarding the processes of assessing capacity and gaining consent for research in the ambulance setting that require further investigation to produce recommendations or guidelines that are specific to ambulance based research. These have implications for ambulance staff, services, researchers and ethics committees who need to consider and assess these issues. Future research will seek to gain the opinions of ethics committee members, as well as a wider group of ambulance service staff, patients and their relatives, and researchers, in order to produce training and guidance that is specific and relevant to ambulance based research.

## Conclusion

This interview study of expert informants offers insight into the challenges of undertaking research in the ambulance setting. This is a major area of growth in clinical research exploring the potential for early intervention and alternative healthcare pathways, in order to improve patient outcomes and reduce costs. We found that there was general consensus among participants about where these challenges lie. Whilst there were different opinions on assessment of capacity and viable consent procedures, participants largely agreed that the production of ethical training and guidance specific to ambulance based research would help researchers, paramedics and ethics committees to navigate these challenges.

## Data Availability

The datasets used and/or analysed during the current study are available from the corresponding author on reasonable request.

## Footnotes

1 This participant, however, did advise against the use of the term ‘assent’ to describe this process, as it does not appear in UK legislation.

2 The National Research Ethics Service (NRES) has been replaced and is currently a core function of the Health Research Authority (HRA) in the UK.

3 They illustrated this point as follows: “It’s a little bit like organ donation, you can be generally supportive of the concept but you don’t like to face the idea on an individual basis because you are then looking your own mortality in the face as it were” (EX13).

4 Note that the Mental Capacity Act (2005) applies to England and Wales. The equivalent legislation in Scotland is the Adults with Incapacity (Scotland) Act (2000). The clinical trials regulations apply to the whole of the UK.

5 The amendment to the Medicines for Human Use (Clinical Trials) Regulations 2006 allows participants who lack capacity and where a legal representative is unavailable to be enrolled on to a the trial without consent, where the Research Ethics Committee has approved this method of enrolment.

## References

[1] Roberts I, Prieto-Merino D, Shakur H, Chalmers I, Nicholl J (2011). Effect of consent rituals on mortality in emergency care research. Lancet.

[2] Dickert NW, Scicluna VM, Baren JM, Biros MH, Fleischman RJ, Govindarajan PR (2015). Patients’ perspectives of enrollment in research without consent: the patients’ experiences in emergency research-progesterone for the treatment of traumatic brain injury study*. Crit Care Med.

[3] Biros MH (2016). Never, always and maybe. Addressing attitudes of patients towards emergency medicine research. Emerg Med J.

[4] Ankolekar S, Parry R, Sprigg N, Siriwardena AN, Bath PMW. Views of Paramedics on Their Role in an Out-of-Hospital Ambulance-Based Trial in Ultra-Acute Stroke: Qualitative Data From the Rapid Intervention With Glyceryl Trinitrate in Hypertensive Stroke Trial (RIGHT). Annals of Emergency Medicine. 2014;64(6):640–8. doi:10.1016/j.annemergmed.2014.03.016. 10.1016/j.annemergmed.2014.03.016 24746844

[5] Turner J. Building the evidence base in pre-hospital urgent and emergency care. A revie of research evidence and priorities for future research. In: Health Do, editor. London 2010.

[6] Burges Watson DL, Sanoff R, Mackintosh JE, Saver JL, Ford GA, Price C (2012). Evidence from the scene: paramedic perspectives on involvement in out-of-hospital research. Ann Emerg Med.

[7] Goldkind SF, Brosch LR, Biros M, Silbergleit R, Sopko G (2014). Centralized IRB models for emergency care research. IRB: Ethics & Human Research.

[8] Thompson J (2003). Ethical challenges of informed consent in prehospital research. CJEM: Canadian Journal of Emergency Medicine.

[9] Armstrong S, Langlois A, Laparidou D, Dixon M, Appleton JP, Bath PM (2017). Assessment of consent models as an ethical consideration in the conduct of prehospital ambulance randomised controlled clinical trials: a systematic review. BMC Med Res Methodol.

[10] Chin TL, Moore EE, Coors ME, Chandler JG, Ghasabyan A, Harr JN (2015). Exploring ethical conflicts in emergency trauma research: the COMBAT (control of major bleeding after trauma) study experience. Surgery..

[11] Morgans A (2010). Waiver of informed consent in Prehospital emergency Health Research in Australia. Monash Bioethics Review.

[12] Sims CA, Isserman JA, Holena D, Sundaram LM, Tolstoy N, Greer S (2013). Exception from informed consent for emergency research: consulting the trauma community. J Trauma Acute Care Surg.

[13] Goldstein JN, Delaney KE, Pelletier AJ, Fisher J, Blanc PG, Halsey M (2008). A brief educational intervention may increase public acceptance of emergency research without consent. J Emerg Med.

[14] Harron K, Woolfall K, Dwan K, Gamble C, Mok Q, Ramnarayan P (2015). Deferred consent for randomized controlled trials in emergency care settings. Pediatrics..

[15] Buckley JM, Irving AD, Goodacre S (2016). How do patients feel about taking part in clinical trials in emergency care?. Emerg Med J.

[16] Davies H, Shakur H, Padkin A, Roberts I, Slowther A-M, Perkins GD. Guide to the design and review of emergency research when it is proposed that consent and consultation be waived. Emerg Med J. 2014. 10.1136/emermed-2014-203675. 10.1136/emermed-2014-203675 25082718

[17] Kasner SE, Baren JM, Le Roux PD, Nathanson PG, Lamond K, Rosenberg SL et al. Community Views on Neurologic Emergency Treatment Trials. Annals of Emergency Medicine. 2011;57(4):346–54.e6. doi:10.1016/j.annemergmed.2010.07.009. 10.1016/j.annemergmed.2010.07.009 20875693

[18] Gray JD (2001). The problem of consent in emergency medicine research. Canadian Journal of Emergency Medicine.

[19] Biros MH, Dickert NW, Wright DW, Scicluna VM, Harney D, Silbergleit R (2015). Balancing ethical goals in challenging individual participant scenarios occurring in a trial conducted with exception from informed consent. Acad Emerg Med.

[20] Dickert NW, Mah VA, Baren JM, Biros MH, Govindarajan P, Pancioli A et al. Enrollment in research under exception from informed consent: The Patients’ Experiences in Emergency Research (PEER) study. Resuscitation. 2013;84(10):1416–1421. doi:10.1016/j.resuscitation.2013.04.006. 10.1016/j.resuscitation.2013.04.006 PMC3770787 23603291

[21] The Medicine for Human Use (Clinical Trials) Regulation, (2004).

[22] The Mental Capacity Act, (2005).

[23] GMC. Good practice in research and Consent to research. London: General Medical Council2013.

[24] Hargreaves K, Goodacre S, Mortimer P (2014). Paramedic perceptions of the feasibility and practicalities of prehospital clinical trials: a questionnaire survey. Emerg Med J.

[25] McClelland G (2013). The research paramedic: a new role. Journal of Paramedic Practice.

[26] Shaw L, Price C, McLure S, Howel D, McColl E, Younger P (2014). Paramedic initiated Lisinopril for acute stroke treatment (PIL-FAST): results from the pilot randomised controlled trial. Emerg Med J.

[27] Biros MH. Does Community Consultation Matter? Academic Emergency Medicine. 2013;20(1):104–5. doi:doi:10.1111/acem.12044. 10.1111/acem.12044 23570484

[28] Hennink MM, Kaiser BN, Marconi VC (2017). Code saturation versus meaning saturation:how many interviews are enough?. Qual Health Res.

[29] Nelson MJ, DeIorio NM, Schmidt TA, Zive DM, Griffiths D, Newgard CD (2013). Why persons choose to opt out of an exception from informed consent cardiac arrest trial. Resuscitation..

[30] Dickert NW, Govindarajan P, Harney D, Silbergleit R, Sugarman J, Weinfurt KP (2014). Community consultation for Prehospital research: experiences of study coordinators and principal investigators. Prehospital Emergency Care.

[31] Sadler GR, Lee H-C, Lim RS-H, Fullerton J. Research Article: Recruitment of hard-to-reach population subgroups via adaptations of the snowball sampling strategy. Nursing & Health Sciences. 2010;12(3):369–74. doi:doi:10.1111/j.1442-2018.2010.00541.x. 10.1111/j.1442-2018.2010.00541.x PMC3222300 20727089

